# Training-of-Trainers Neuroscience and Mental Health Teacher Education in Liberia Improves Self-Reported Support for Students

**DOI:** 10.3389/fnhum.2021.653069

**Published:** 2021-06-18

**Authors:** Kara Brick, Janice L. Cooper, Leona Mason, Sangay Faeflen, Josiah Monmia, Janet M. Dubinsky

**Affiliations:** ^1^Carter Center Mental Health Program, Monrovia, Liberia; ^2^Peace Corps Liberia, Monrovia, Liberia; ^3^Ministry of Education, Monrovia, Liberia; ^4^Department of Neuroscience, University of Minnesota, Minneapolis, MN, United States

**Keywords:** mental health literacy, neuroeducation, teacher professional development, stigma reduction, trauma-informed teaching

## Abstract

Education programs have been central to reestablishing social norms, rebuilding public educational institutions, and addressing public attitudes toward mental illness in Liberia following a protracted civil war and the Ebola epidemic. The aim of this study was to determine if a program combining an understanding of neuroscience with mental health literacy content could increase teachers’ awareness of students’ mental health issues and produce changes in teacher attitudes and classroom practices. A tiered Training-of-Trainers approach was employed. The first workshop trained 24 Liberian secondary science teachers in the neurobiology of learning, memory, emotions, stress and adolescent brain development. A Leadership Team formed from eight of the Tier I participants then adapted the curriculum, added in more mental health literacy content and led four Tier II workshops and four follow-up Refresher sessions. Participants completed a neuroscience knowledge test and surveys assessing stigma, general perceptions of people with mental illness, and burnout. A subset of Tier II teachers participated in a structured interview at the Refresher time point. Teachers in both tiers acquired basic neuroscience knowledge. Tier I, but not Tier II teachers significantly improved their surveyed attitudes toward people with mental illness. No changes were found in overall teacher burnout. Despite these survey results, the interviewed Tier II teachers self-reported behavioral changes in how they approached their teaching and students in their classrooms. Interviewees described how they now understood social and emotional challenges students might be experiencing and recognized abnormal behaviors as having a biopsychosocial basis. Teachers reported reduced use of verbal and corporal punishment and increased positive rewards systems, such as social and emotional support for students through building relationships. Refresher discussions concurred with the interviewees. In contrast to previous teacher mental health literacy programs which did not bring about a change in helping behaviors, this pilot program may have been successful in changing teacher knowledge and self-reported behaviors, improving teacher–student relationships and decreasing harsh discipline. The combination of basic neuroscience concepts with training on how to recognize mental health issues and refer students should be investigated further as a strategy to promote teacher mental health literacy.

## Introduction

A number of programs have been developed in Liberia as part of a concerted attempt to construct a system of care for mental health (MH). MH needs remain high among a population recovering from both civil war (1989–2003) and the Ebola outbreak (2013–2015). These programs raise awareness of MH issues among the public, train professionals and persons living with MH problems, and provide early intervention strategies (Kohrt et al., 2015, 2018; Ministry of Health, 2016; Gwaikolo et al., 2017; Liberia Center for Outcomes Research in Mental Health, 2020). To provide diagnostic and support services in a country with only a few psychiatrists, a cadre of Mental Health Clinicians (MHCs) has been trained to diagnose and treat people with mental illness (MI) (Gwaikolo et al., 2017; Kohrt et al., 2018). These MHCs represent a service that people were unaccustomed to utilize. To support prevention and early intervention, the Government of Liberia identified schools as a locus of health and health education and teachers as sources for accurate MH education, initial case finding, and referral support for students in need of MH services (Ministry of Health, 2016; Gwaikolo et al., 2017; Patel et al., 2018). In some schools MHCs are available on site or by referral.

Education is recognized as an important proximal factor for ameliorating the negative effects of personal, social and global traumas contributing to mental health problems (Patel et al., 2018). Social-emotional learning programs in schools constitute important community level practices for improving awareness of issues related to mental, neurological and psychiatric illnesses in low and middle income countries (LMIC) (Petersen et al., 2016). School based intervention programs have improved MH knowledge and to a lesser extent help-seeking but changes in attitudes did not necessarily accompany knowledge gains (Salerno, 2016). In Brazil, teachers were successfully trained to recognize and refer students with social, mental and behavioral problems (Vieira et al., 2014). A social contact-based approach, where individuals share their stories of struggling with MI and subsequent recovery, has been applied for organizations supporting people living with mental illness in Liberia, as this approach is most effective with adult audiences (Corrigan et al., 2012; Gwaikolo et al., 2017). An educational approach has been more effective with youth audiences, who, like the Liberian teachers, have had less exposure to the biopsychosocial model of MI and the neuroscience that supports this model (Corrigan et al., 2012), further influencing this choice.

Teachers are prominent among the community level resources needed to provide developmental support for resilience among youth (Luthar et al., 2015). Children with histories of exposure to war, violence and other physical and emotional trauma can often behave in ways incompatible with expected classroom behavior (NCTSN Core Curriculum on Childhood Trauma Task Force, 2012). One way to indirectly benefit children’s resilience and mental health is to promote the knowledge and well-being of their teachers in order to strengthen these caregivers’ ability to promote positive relationships with students (Luthar et al., 2015). Teacher support is among the community level variables more amenable to change to build systems that positively impact children’s resilience (Luthar et al., 2015; Ministry of Health, 2016). In an international review of MH literacy programs for teachers, increases in MH knowledge and reductions in stigma were widely reported (Yamaguchi et al., 2020). A MH literacy training program in Malawi and Tanzania significantly increased teachers’ knowledge of MH issues and improved their attitudes toward MH (Kutcher et al., 2015, 2016). Teacher MH trainings typically addressed MH signs, symptoms and how to respond (Yamaguchi et al., 2020). A few programs also included behavioral information on child development (Pacione and Cooper, 2014; Anderson et al., 2019). These programs, of limited duration – from a few hours to a day or so, left the audience requesting more training, and produced little evidence of change in teacher helping behaviors (Anderson et al., 2019).

Individuals with MI also face social consequences within educational settings. People with MI are viewed as abnormal and incompetent, suffering both stigma and lowered social status (Phelan et al., 2018). Within the school environment, affected youth perceive stigma against persons with MI from teachers, staff and peers (Moses, 2010; Bowers et al., 2013). Student achievement is influenced by teacher expectations. Low teacher expectations, often as a consequence of student behaviors, act as a self-fulfilling prophecy for low attainment. Stigmatized groups are more vulnerable to this effect (Jussim and Harber, 2005; Timmermans et al., 2018). The presence of MI and the associated externalizing or internalizing behaviors is generally associated with lowered overall academic attainment, although this does not hold for all diagnoses (McLeod et al., 2012; Dalsgaard et al., 2020). MI does not, *a priori*, preclude an ability to achieve academically (McLeod et al., 2012; Dalsgaard et al., 2020). This link between academics and social behavior remains an association and not a causal relationship (Algozzine et al., 2011; McLeod et al., 2012; Dalsgaard et al., 2020); yet teachers rate students with known emotional problems as having inadequate academic performance (Algozzine et al., 2011). When controlling for academic aptitude and for the co-occurrence among MH and behavior problems, the presence of a MH issue does not necessarily predict lowered academic outcomes, suggesting that negative educational outcomes may not result from inherent traits but rather from the social response of the schools to the presence of these problems (Algozzine et al., 2011; McLeod et al., 2012). In recent years, positive behavioral interventions and support programs have begun to ameliorate the behavioral issues and possibly improve educational outcomes (Patel et al., 2018; Sugai and Horner, 2020). School based MH literacy programs provide a mechanism for addressing both teacher expectations and needed student supports (Yamaguchi et al., 2020).

Neuroscience provides both a framework for understanding how traumatic experiences can reshape learning and relationships in a child’s world and a hopeful prospect that brain plasticity nurtured in a safe school environment can produce resilience (NCTSN Core Curriculum on Childhood Trauma Task Force, 2012; Howard, 2019). Understanding the developmental neurobiology of children’s brains, a core concept essential for dealing with traumatic stress responses in childhood (NCTSN Core Curriculum on Childhood Trauma Task Force, 2012), may provide an engaging entry point for raising teachers’ awareness of MH issues among students. A neurobiological approach was envisioned as a way to expose teachers to a biopsychosocial model of mental health, to recognize their role in supporting children with social-emotional and behavioral challenges, and to dispel myths and misconceptions surrounding mental illnesses and epilepsy (Gwaikolo et al., 2017; Jones and Kahn, 2017). This knowledge should support efforts to ameliorate or modify student behaviors so they remain in school and excel academically (Gwaikolo et al., 2017; Jones and Kahn, 2017). Understanding brain plasticity is a component in interventions promoting growth mindsets, which teach how to apply the core ideas of brain plasticity to personal achievement, response to rejection, or conflict (Blackwell et al., 2007; Yeager et al., 2013, 2019). Such programs have demonstrated positive impacts upon student world view, academic performance and prosocial behaviors in the face of adversity (Blackwell et al., 2007; Yeager et al., 2013, 2019). Educating teachers in neuroscience has resulted in adoption of more student-centered and active-learning pedagogies, improved classroom cognitive environments and more social and emotional support for students (Dubinsky et al., 2013; Dubinsky et al., 2019). In the United States, school-based mental health awareness interventions have resulted in increased knowledge, acceptance, and help seeking behaviors among adolescents (Salerno, 2016). Students’ social and emotional well-being is integrally tied to their cognitive, linguistic and academic development (Jones and Kahn, 2017). Increases in pro-social behaviors and decreases in risky behaviors and MH issues accompany positive student-teacher relationships, an increased sense of belonging, connectedness, and students’ feelings of safety and being cared for Aldridge and McChesney (2018). Warm and engaging teacher–student relationships are related to greater student learning (Jones and Kahn, 2017).

### Objectives

We therefore designed a training program for Liberian teachers focused upon the foundational concepts that neuroscience brings to both education and the etiology of mental disorders. For a science teacher audience, an education approach was chosen as a starting point. In an effort to maximize resources, a Training-of-Trainers approach (Kohrt et al., 2015) was employed; teachers from the Tier I workshop were expected to lead subsequent workshops for Tier II teachers. The Training-of-Trainers approach has been used successfully in training teachers in Malawi and Tanzania to use a MH awareness classroom curriculum (Kutcher et al., 2015, 2016). Table 1 provides goals for the program and the alignment of various programmatic standards with those goals and program elements. Program evaluation focused upon the success of meeting those goals and upon the success of the training approach. Program outcomes regarding teachers affective and motivational attitudes and pedagogy (goals 2 and 3) and the translation from Tier I to Tier II appear in a companion paper (Brick et al., 2021). Here the focus is on teachers’ acquisition of neuroscience knowledge (goal 1) and development of their understanding of MH issues in students (goal 4).

**TABLE 1:** Alignment of program goals and elements with various standards.

Program goal or other element	Tier I activity	Tier II activity	Standards for effective PD	Pedagogical practices developing countries	Lessons for project design in LMIC	Ministry of education goals
Goal 1: Understanding the basic neuroscience of brain function and dysfunction associated with neurological and mental disorders	Lectures (∼20% of total time)	Lectures (∼50% of total time)	Content focus	Sustained attention; Frequent and relevant use of learning materials beyond the textbook	Integrated set of mutually reinforcing activities	Production and implementation of a curriculum that is relevant, appropriate and addresses major content and quality concerns
Goal 2: Modeling student-centered teaching practices	Classroom activities, model building, experiments, discussions	Classroom activities, model building, experiments, discussions	Active learning, collaboration, use of Models	Drawing on students’ backgrounds and experiences; flexible use of whole-class, group and pair work where students discuss a shared task; open and closed questioning, expanding responses, encouraging student questioning; demonstration and explanation, drawing on sound pedagogical content knowledge	Integrated set of mutually reinforcing activities	
Goal 3: Improving the pedagogical expertise and confidence of teachers	Reflection, discussion, role play and practice teaching	Reflection, discussion, role play and practice teaching	Coaching and expert support, feedback and reflection	Feedback, inclusion	Integrated set of mutually reinforcing activities	
Goal 4: Incorporating a biopsychosocial model of mental health in the teacher mindset	Discussion	Activities from the Good Schools Toolkit*; videos, discussions		Creating a safe environment in which students are supported in their learning; inclusion	Integrated set of mutually reinforcing activities	To make those provisions and arrangements that result in the school environment being clean, sanitary, violence-free and sufficiently conducive for all students, especially girls, to feel safe and at ease
Tiered training	Trained by visiting neuroscientist	Trained by Tier I leadership team	Coaching and expert support, feedback and reflection	Administrative and peer support	Wide engagement of stakeholders at all levels; capacity building	Development and implementation of an in-service program to upgrade and update trained teachers
Evaluation	Daily reflections; knowledge test; surveys	Daily reflections; knowledge test; surveys; interviews	Feedback and reflection		Monitoring and evaluation	
Duration	10 days	5 + 2 days	Sustained duration		Appropriate timeframes; sufficient resources for implementation	
Reference	Brick et al., 2021	*Devries et al., 2015, Brick et al., 2021	Darling-Hammond et al., 2017	Westbrook et al., 2013	Lehner, 2012	Ministry of Education, 2010

Teachers were expected to benefit from exposure to an understanding of the neuroscience of learning and memory, and active learning pedagogy (Dubinsky et al., 2019). The neuroscience knowledge was expected to connect with their prior knowledge, to influence both teaching practices and understanding of mental health etiology, and to eventually be transferred to students. We hypothesized that teachers would develop a view that students with social, emotional and behavior issues had the potential to succeed in the educational system. However, we did not know *a priori* if measures of stigma would change since this topic was not specifically taught. This pilot study employed a mixed methods approach using both qualitative and quantitative methods to analyze how Liberian science teachers responded to an experimental curriculum in which basic neuroscience was taught as a means for improving teacher understanding of student mental health issues. The specific research questions were:

(1) How well did Tier I and Tier II teachers learn neuroscience?
(2) How did both Tier I and Tier II trainings alter teacher attitudes toward the mental health issues of their students?
(3) How did teachers adapt their teaching to accommodate any new understanding of student mental health?

## Materials and Methods

### Study Design

The current exploratory study tests if an existing intervention, training teachers in neuroscience (MacNabb et al., 2006b; Roehrig et al., 2012; Schwartz et al., 2019), can produce previously untested outcomes regarding teachers’ attitudes toward the MH needs of their students. Alternatively, this study could be considered at the design and development stage as the intervention model is being expanded to include the training-of-trainers locally in Liberia (NSF, 2013). This study builds on evidence-based and evidence-informed knowledge and practices to investigate the applicability and consequences of training Liberian teachers in neuroscience. Although not perfectly aligned with this work, the SQUIRE-EDU, GREET, and SRQR guidelines have informed preparation of this report (O’Brien et al., 2014; Phillips et al., 2016; Ogrinc et al., 2019).

A mixed methods approach was used to assess the efficacy of a two tiered PD program for Liberian secondary science teachers combining neuroscience and mental health. Quantitative survey data was collected from teachers in both tiers regarding their knowledge of neuroscience and their attitudes toward people with mental illness. Qualitative data from structured interviews was collected from a subset of Tier II teachers on how they applied this knowledge in their classrooms.

### Intervention

This pilot project trained a set of Liberian secondary science teachers in the neurobiology of learning and memory, emotional processing and stress, and the etiology of epilepsy and PTSD. In this two-tiered training, a visiting neuroscientist with prior experience providing teachers with neuroscience training (MacNabb et al., 2006b; Roehrig et al., 2012; Dubinsky et al., 2013, 2019; Schwartz et al., 2019) delivered a workshop (10 days over 2 weeks) for Liberian secondary science teachers (Tier I). A subset of the Tier I teachers then adapted the material for the Liberian context and delivered a series of 1-week (5 full day) workshops to train additional Tier II Liberian teachers [see below, Table 1 and (Brick et al., 2021)]. An intensive training approach was chosen because short neuroscience exposures change very little (Howard-Jones et al., 2020) and evaluation of United States science teacher training programs determined 80 or more hours of PD were needed for teachers to enact substantial classroom changes (Lawrenz et al., 2007). The initial Tier I training occurred in August 2018, modeled after a successful program developed for United States teachers (MacNabb et al., 2006b; Roehrig et al., 2012; Dubinsky et al., 2013, 2019; Schwartz et al., 2019). Lessons plans and resources used in the workshop were drawn mainly from open internet neuroscience resources (MacNabb et al., 2006a; SFN, 2019), as recommended for educational improvement in Sub-Saharan Africa (Wolfenden et al., 2012).

A subset of eight Tier I teachers joined staff to form a Leadership Team that planned and delivered four Tier II trainings in October through December 2018. The Leadership Team selected the Tier II neuroscience content and added mental health awareness content and how to identify early warning signs of MH issues from the Manual of School Mental Health (Pacione and Cooper, 2014) and The Good Schools Toolkit (Section 3.5, Devries et al., 2015), resources specific to successful MH education in Africa. Additional internet resources were used to stimulate discussions of students’ and teachers’ MH issues (Brick et al., 2021). The Tier II neuroscience content was reviewed by email and approved by the visiting neuroscientist. Tier II content was approximately 50% similar to Tier I content (see Brick et al., 2021 for a more detailed discussion of workshop content and fidelity). Workshop delivery consisted of Liberian teachers training fellow Liberians. Program staff on the Leadership Team guided the team in setting standards for and maintaining content quality. In response to teacher requests for support, a series of four 2-day follow-up Refreshers were organized by the Leadership Team in March and April, 2019. On 1 day, Tier II teachers shared stories about and reflected on their teaching after the workshop and had a digital question and answer session with the visiting neuroscientist. On the other day of the Refreshers, teachers practiced teaching neuroscience lessons in local secondary schools. All sessions, except for this one Refresher day, were held in neutral meeting spaces outside of a school setting. All of these processes received support from the Ministry of Education and were overseen and hosted by an international non-governmental organization, as recommended for successful education projects in LMIC (Lehner, 2012). All events were held in Monrovia, Liberia except for one Tier II workshop and one Refresher which were held in Kakata, a small city interfacing with more rural areas of Liberia.

### Participants

Secondary science teachers were chosen as the target audience because they taught to the Liberian and West African science standards that included biology and some neuroscience knowledge (W.E.A.C, 2011). In keeping with Liberian traditions and as a gesture of respect and partnership, the Ministry of Education Office of Science Education called Principals and Heads of high schools within the Monrovia Consolidated School System and Kakata Government Schools and asked them to choose one or two science teachers to send for training. This non-random process ensured that participants had appropriate backgrounds and positions (Best and Kahn, 2006). The Tier I workshops were attended by 15 high school science teachers, one university level instructor, and eight staff members from the Ministry of Education. Ninety-two science teachers attended the Tier II workshops. Participants only received compensation for transportation expenses; they were not paid for their time or effort. The majority of participants (63% Tier I, 53% Tier II) were from Montserrado County, which contains the Liberian capital of Monrovia where most of the workshops were held. Females are underrepresented among Liberian teachers (Stromquist et al., 2013) and among participants (38% Tier I, 26% Tier II). For both tiers, 63% of the teachers held BS degrees. However, 35% of Tier II teachers were without university level teacher training as opposed to 21% of Tier I participants. Teachers’ educational backgrounds have been previously reported (Brick et al., 2021). Only one teacher in each tier had any prior MH training (Brick et al., 2021).

Participation in the workshops and in the data gathering were described to participants as separate processes. All participants voluntarily and formally consented on day 1 to be a part of the workshop outcome study, including to be interviewed, conducted according to IRB protocols approved separately by the University of Liberia and Emory University. Teachers were assigned alphanumerical identifiers to use instead of their names on all surveys and assessments.

### Instruments

Program outcomes were measured through a mixed-methods approach that included participant daily reflections, pre-post workshop multiple choice knowledge tests, self-reported surveys and interviews. For the 24 Tier I participants, a 13 question knowledge test was administered on the first and last days of the workshop (MacNabb et al., 2006b). The Tier I survey was administered at the end of the workshop twice, once regarding how teachers felt at that time, and once projecting how they remembered feeling prior to taking the workshop. Thus a retrospective pre-survey approach was taken where respondents understand what their true initial knowledge was after they have become fluent in the provided content (Levinson et al., 1990; Bhanji et al., 2012). For Tier II, survey data were collected at three time points, on the first and last days of the weeklong workshops and at the Refreshers. Teachers in one Tier II workshop completed a shorter 4 question neuroscience knowledge test tailored to the Tier II content. Another subset of 10 Tier II teachers were interviewed by two Leadership Team members at one Refresher. The Tier II interviewees were determined by convenience and were not randomly selected. Field notes were kept for the discussions among teachers at the Refreshers.

Survey instruments were chosen to measure the impact of the Brain Science training on participants’ knowledge of neuroscience (MacNabb et al., 2006b) and attitudes toward mental health issues. Scales employed for these attitudes included a version of the Social Distance scale to measure MH stigma in Liberia (Kohrt et al., 2018; Boazak et al., 2019), and a General Perceptions of People with Mental Illness scale (Kohrt and Swaray, 2011), both in Liberian English. The Social Distance scale consisted of nine statements regarding willingness to engage with someone with mental illness and uses a 4 point Likert scale of 1, definitely willing; 2, probably willing; 3, probably unwilling; and 4, definitely unwilling for a range of 9–36. The General Perceptions scale consisted of seven statements regarding ideas about or attitudes toward people with mental illness and uses a 4 point Likert scale of 1, strongly disagree to 4, strongly agree, for a range of 7–28. For both scales, lower scores indicate less prejudice. The Maslach Burnout Inventory for Educators (Maslach et al., 1996) was also included to determine if the neuroscience trainings effected burnout. This scale consisted of 22 statements expressing stress and uses a Likert scale for frequency of encountering these feelings of 0, never to 6, every day, for a range of 0–132. Lower numbers indicate less stress. Permission was granted for use of these scales.

The structured interview questions appear in the Supplementary Materials.

### Quantitative Analysis

All surveys employed Likert scales. After reverse coding appropriate items, survey responses were summed across an entire scale and then averaged across all participants at a single time point. While Tier I and II responses were kept separate, responses across all of the Tier II workshops were aggregated. Tier I comparisons between post and retrospective pre time points were made using two tailed *t*-tests (Graphpad Prism, version 6.1). For Tier II comparisons among pre, post and Refresher time points, data were analyzed using one-way ANOVAs with Tukey’s multiple comparison post-tests (Graphpad Prism, version 6.1). No data were discarded.

### Qualitative Analysis

In-depth individual interviews were conducted in Liberian English based on a structured interview guide. These interviews were conducted at the end of one Refresher workshop with 10 participants who were teaching in the Liberian school system and who attended Tier II trainings. The interview questions explored the role of the teachers, changes and challenges in their teaching since the training, student behavior, mental health issues in the classroom, and feedback on the trainings. All interviews were digitally recorded and subsequently transcribed and annotated. Interviewers also wrote field notes.

Interviews were initially coded using NVivo 12. A framework analysis approach was applied as it was most appropriate for this multidisciplinary project and could be applied inductively without forcing a particular theoretical perspective (Gale et al., 2013). A codebook was developed after an iterative reading of the interviews to generate codes (SKF). Additional codes based on the study objectives were added to the codebook. Two authors (KB and JMD) separately coded all interviews, using the field notes for context. Three authors (KR, JMD, and JLC) then iteratively discussed and recoded the data until consensus was reached. Independently, two authors (KB and JMD) summarized the coded data in written form. These summaries were subsequently discussed among the three authors (KR, JMD, and JLC), edited and reworked until themes emerged and consensus was reached.

## Results

### Knowledge of Neuroscience

**FIGURE 1:**
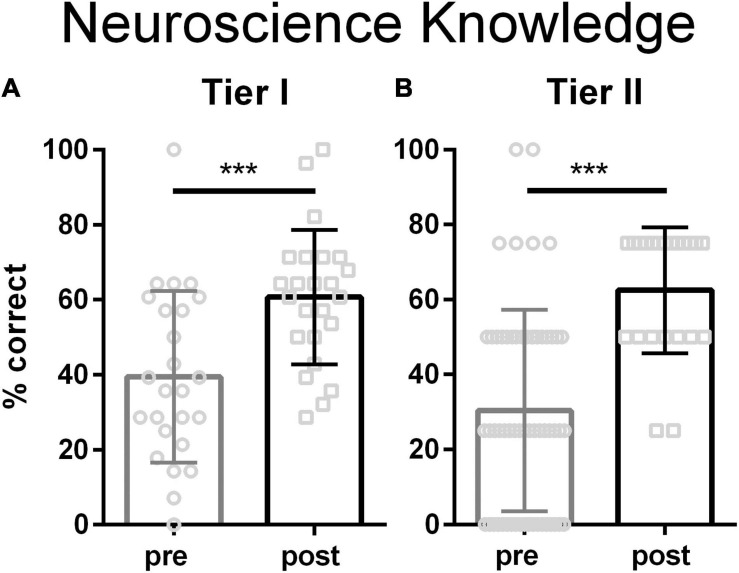
Knowledge of neuroscience for teachers attending Tier 1 **(A)** and Tier II **(B)**. Tier I teachers took a 13 question multiple choice test (MacNabb et al., 2006b). Tier II teachers took a 4 question multiple choice test. Both Tier I and Tier II teachers significantly increased their knowledge of the neurobiology of learning and memory (Tier I, *p* = 0.0007, Cohen’s *d* = 1.0; Tier II, *p* < 0.0001, Cohen’s *d* = 1.3; 2 tailed *t-*tests). Bars are mean ± stdev; Tier I *N* = 25 pre, 24 post, Tier II *N* = 60 pre, 22 post. *** represents *p* < 0.001.

Both Tier I and Tier II teachers’ knowledge of neuroscience increased significantly after their respective workshops (Figure 1). Tier I teachers took a knowledge test equivalent to that used in prior trainings and scores improved comparably (MacNabb et al., 2006b). Since the content and length of the Tier II workshop was less than that of the Tier I workshop, Tier II teachers took a shorter knowledge test focusing only on brain plasticity. The plasticity concepts were grasped by all attendees.

### Attitudes Toward Mental Health

**FIGURE 2:**
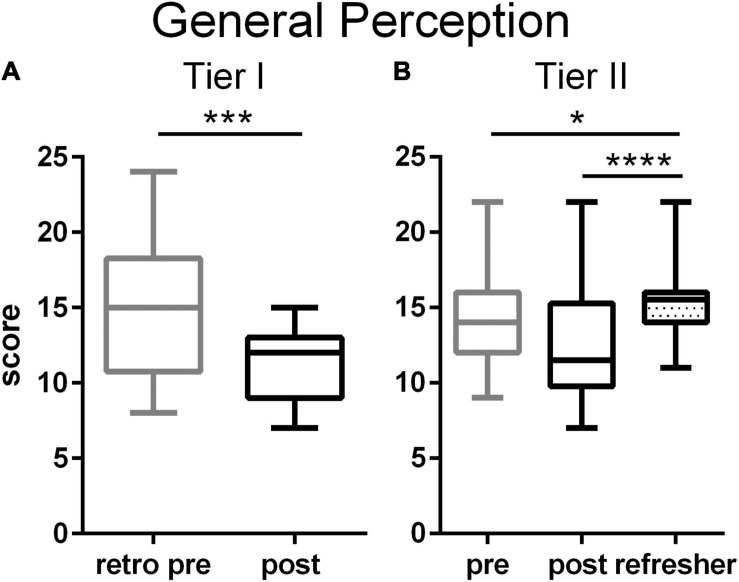
General Perceptions of people with MI among Tier I **(A)** and Tier II **(B)** teachers. Tier I teachers significantly reduced negative attitudes toward people with mental illness after the training (*p* = 0.0007, Cohen’s *d* = –1.09, two tailed *t*-test). Among the Tier II teachers, negative attitudes declined only as a trend after the training, but rose significantly at the time of the Refresher (*p* < 0.0001, η^2^ = 0.15, one way ANOVA with Tukey’s multiple comparison post-test). Boxes represent 25th to 75th percentiles with an internal bar at the median. Whiskers delineate maximum and minimum data points. Tier I *N* = 22 retrospective pre, 23 post, Tier II *N* = 44 pre, 38 post, 56 Refresher. For the post tests, *, ***, **** represent *p* < 0.05, 0.001, 0.0001, respectively.

Initially participants in both tiers scored in the middle of the range on the General Perception of people with mental illness survey scale. These scores significantly decreased at the end of the workshop for Tier I teachers (Figure 2A and Supplementary Materials), indicating fewer misperceptions. At the end of the Tier II workshop, the median score moved in the correct direction, but not significantly so (Figure 2B). At the Refresher time point, Tier II teachers’ ratings on this scale were significantly greater than both initial and post-workshop ratings.

**FIGURE 3:**
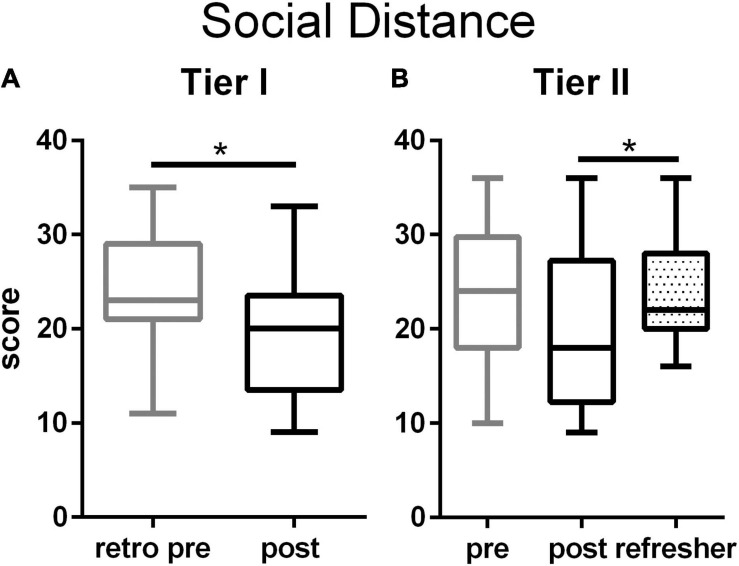
Social distance scale among Tier I **(A)** and Tier II **(B)** teachers. Tier I teachers significantly reduced negative attitudes toward people with mental illness after the training (*p* = 0.020, Cohen’s *d* = –0.70, two tailed *t*-test). Among the Tier II teachers, social distancing attitudes declined only as a trend after the training, but rose significantly at the time of the Refresher (*p* = 0.031, η^2^ = 0.049, one way ANOVA with Tukey’s multiple comparison post test). Boxes represent 25th to 75th percentiles with an internal bar at the median. Whiskers delineate maximum and minimum data points. Tier I *N* = 23 retrospective pre, 24 post, Tier II *N* = 44 pre, 40 post, 56 Refresher. For the post tests, *represents *p* < 0.05.

On the Social Distancing Scale, teachers in both tiers initially scored above midrange. The training significantly decreased Tier I teachers’ negative attitudes toward people exhibiting neurodiverse behaviors (Figure 3A and Supplementary Materials). Among Tier II teachers, the training did not significantly alter ratings on this scale (Figure 3B). By the Refresher time point, the variability among Tier II teachers’ ratings had decreased, resulting in a significant rise in negative attitudes compared to the end of the workshop. Refresher time point ratings were, however, not significantly different from the initial time point.

**FIGURE 4:**
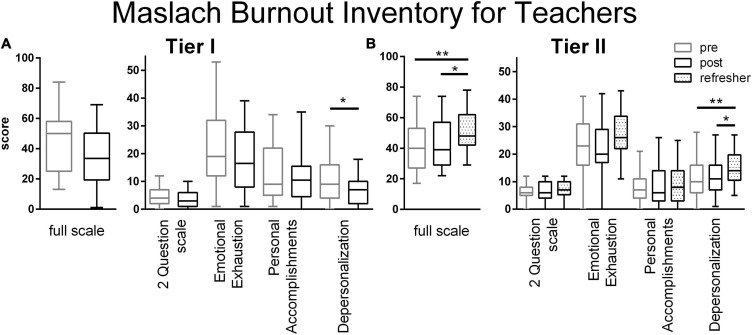
Maslach Burnout Inventory Scale ratings from Tier I **(A)** and Tier II **(B)** teachers. Decreases in burn out scores were only observed in Tier I teachers after the training on the Depersonalization subscale (*p* = 0.061, full scale; *p* = 0.269, Two Question Scale; *p* = 0.141, Emotional Exhaustion Scale; *p* = 0.457, Personal Accomplishments Scale; *p* = 0.041, Cohen’s *d* = –0.61, Depersonalization Scale; two tailed *t*-test). Among the Tier II teachers, burn out ratings increased by the Refresher (full scale, *p* = 0.0019, η^2^ = 0.088; Two Question Scale, *p* = 0.437; Emotional Exhaustion Scale, *p* = 0.0.038, η^2^ = 0.047; Personal Accomplishments, *p* = 0.615; Depersonalization, *p* = 0.0025, η^2^ = 0.085; one way ANOVA with Tukey’s multiple comparison posttest). Boxes represent 25th to 75th percentiles with an internal bar at the median. Whiskers delineate maximum and minimum data points. Tier I *N* = 23 retrospective pre, 24 post, Tier II *N* = 43 pre, 39 post, 56 Refresher. For the post tests, *, ** represent *p* < 0.05, 0.01, respectively.

For the Maslach Burnout Inventory Scale for Teachers, Tier I teachers’ ratings appeared slightly lower after the training, but only reached significance for the subscale on Depersonalization (Figures 4A,B and Supplementary Materials). The training did not immediately change Tier II teachers’ burnout ratings on the full scale or on any subscale (Figures 4C,D). At the Refresher time point, Tier II teachers’ burn out ratings were significantly higher for the full scale and for the Depersonalization subscale. Other factors during the intervening period between the training and the Refresher may be responsible for this increase in burnout.

### Teacher Interviews

Tier II teachers’ attitudes toward student mental health issues were more clearly expressed in the interviews conducted at the Refresher time point. Three themes described the changes emerging from analysis of these interviews: (1) Teachers understood adolescent development and the causes of student MH issues; (2) Teachers endeavored to build positive relationships with students to address social and emotional issues; and (3) harsh disciplinary practices were abandoned in favor of inclusive practices and positive reinforcement, shifting the power dynamics within the classroom. The field notes from the Refresher discussions with all Tier II survey respondents were reviewed and found to emphasize these same three themes with enthusiastic, candid and varied examples.

#### Recognizing Mental Health Issues

Teachers embraced an understanding of brain development over the adolescent years. Since adolescents appear to have mature bodies, teachers may easily have assumed their mental capacities were also at adult levels. Understanding that brain plasticity and brain growth continued into early adulthood changed teachers’ expectations of students. “Because as a teacher before … I never knew the difference between adult brain development and that of children’s brain development (T9).” This foundation set the stage for a deepened awareness of student problems and mental health issues.

Teachers appreciated that abnormal behavior, either for the group or the individual, should be traced to an issue. Teachers realized that deeper problems may be indicated by the following ongoing behaviors: being withdrawn, sleepy, inattentive, uncooperative, disruptive, rude, disrespectful, or otherwise non-compliant. To recognize MH issues, teachers compared a student to peers or compared a student to their own prior behavior, observed over a longer timeline. As one astute teacher put it: “Until you are smart, until you are really following students, you will not get to know who is developing mental problems. I don’t have to be a medical doctor to know that someone has some emotional problems, some personal issue. When I know my student… I know that this person has not been in such a mood before… I often refer them to a doctor (T9).” Teachers were cognizant that material and social environments outside of class can greatly affect students’ mental state. These factors included poverty, home environment, overwork, lack of a supportive family, fatigue, psychological and general social or emotional issues. “[students may come to school with] some family problems, so they would just come and lay their heads down (T6).”

Teachers realized the stigmatization associated with mental illness or poor scholastic performance did not imply a student was unintelligent, hopeless or ‘bad.’ “Before then we used to focus too much on the brilliant students and consider those ones that still behind or slow in learning. are wasting our time. In fact, nothing good would get out of them. So sometimes we don’t have patience. But since, the reception of this training, my mentality has completely changed (T9).” Rather they now viewed such students with potential to improve once the problem was addressed: “Everybody are equal. Maybe some people, especially for those that have mental problem, you cannot say, ‘hey you move that side! Man, you rude, you’re good for nothing.’ No. What I learn from the training, that everyone has importance and they have their rights, and as a teacher I’m also the one to point you in the right path (T6).”

For the particular problem of epilepsy, teachers embraced explanations for its neurophysiological causes and became willing to include them in the school culture. In Africa, epilepsy could occur as a consequence of head injury, cerebral malaria or tapeworm infections among other causes (DeGiorgio et al., 2004; Klejnstrup et al., 2018). Teachers were more aware of the stigma surrounding epilepsy and the detriment it caused to such students, who suffered from social isolation, not seeking medical attention, and not being assisted when seizing. “I got a student with epilepsy, and the training I got when I went back, I just apply it right away, because we all felt [before the training] that the epilepsy was contagious, so any time it will happen, we. just shy away and look. But I went there, I told the class. in fact, I call the entire school in one of the devotion [assemblies], and we discuss what are some of the help that we can provide and what is epilepsy. We discuss that, and from there, the child is free now. He moves around, and other people go around [with] the child, in the classroom. They don’t feel like, ‘The epilepsy that you have, other people can contract it.’ So I’ve helped in that direction (T7).”

#### Building Teacher–Student Relationships

Once students’ mental health issues were contextualized and defined, teachers were willing to be patient with and give attention to students with psychological, social or emotional issues. Teachers increased their interactions with students, discussing problems, befriending them and generally helping them solve social and emotional problems. Teachers created opportunities for connection with students to provide emotional support, in particular to talk through whatever might be on their minds if they were showing signs of distress. “I interview them and they will give me their inside history. They will better explain what really they are going through, then from there, [I] start to help them. maybe they are traumatized, then I help them by talking to them. If there is something bothering them, explain the situation. Explain the situation, or through this you can help yourself, talk to them, just be able to counsel them (T5).” Teachers discerned a difference between emotional behavioral problems and those caused by underlying social issues. Teachers made efforts to provide social support by providing meals, a place to study and supplies, despite the financial hardship of often late or missing paychecks (Adebayo, 2019; Romero and Sandefur, 2019). “Students like [those in need], I try talking to them, I make myself available to give them something in the morning. Every morning I told them, ‘When you come, just come, strictly in my office, I will give you something.’ Because of that, this particular child always happy in the morning to come (T5).” Teachers’ extra efforts and increased social support strengthened their relationships with students.

Nine out of 10 teachers related stories about helping individual students address a social, emotional or MH problem through personal involvement in providing care, constructing a care strategy with others, or through obtaining medical care. Success stories included clarifying communication in social situations, building relationships to bring students back into a state of learning, or encouraging other educators to move forward in a different way. Teachers were cognizant that MH support differs from other types of support, requiring an underlying relationship, ongoing talking, and more attention. They investigated root social or home causes of student problems. Teachers referred students to guidance counselors, discussed issues with parents, or accompanied students to seek medical or psychological attention. “Then, if it is something serious, really serious, I will try to get in contact with the student’s parents. Then we together can take it from there. If it means that the student has to see a psychiatrist or a doctor, then we can do it that way, because I believe that my responsibility to search a student and not just restricted in the class. … Maybe the parent might not know what is happening. Cause some students are very much afraid of the parents (T9).” Despite getting more than they expected after scratching the surface of students’ aberrant behaviors, these teachers remained committed to working diligently to resolve them.

#### Disciplinary Practices

Corporal punishment is an acknowledged component of classroom discipline in Liberia as well as in other African nations (Antonowicz, 2010). Interviewed teachers explained this historical and cultural legacy: “… because in the African mentality before then our teachers, those that used to teach our fathers and mothers, it was nothing different from slave and master. Yeah. They were harsh to the extent that they used to beat on students like criminals. Even up to now some of our parents got the mark [scars] (T9).” Teachers learned that ‘beating’ can have a detrimental effect on the physical brain: “I got to know that, especially for the little ones, if you slap them behind their head you will cause problems for the brain (T5).” Teachers admitted to having previously beaten, insulted, shamed and discouraged students: “Before the training I used to beat on students especially those slow learners, … also those students who have the habit of making noise in class. … and also I used to discourage them in class (T2).” They acknowledged that they had not allowed students time to think and had used physical or disciplinary actions in response to wrong answers.

The interviewed teachers contrasted their current practices with their admitted prior use of harsh discipline. Previously, teachers said they used practices that may have been emotionally damaging. Teachers attested to now using patience and dialog to respond to noisy or inattentive students rather than physically beating them. “For instance …, as an instructor in a class sometimes a student annoys you, you get angry, you call the student up … and slap the student’s head, which is very wrong. The workshop, the training has made us to understand that such things is wrong and believe me I decided not to practice that both home and in my school that I teach (T1).” In one of the Refresher sessions, male teachers reported they had ceased engaging in forced physical relationships with female students. Teachers reconsidered the language they used with disruptive students, stopped using profanity and insults, and began using encouraging and more inviting approaches: “[before the training,] we may be insulting them and telling them … ‘you are stupid.’ Make sure, I want you to know that child is not stupid. … So, you as a teacher, we need to share love, patience. Create a new avenue and beginning to take more and more how to get inside to them (T10).”

Teachers shifted from presenting themselves as powerful authority figures or managers to becoming more friendly and approachable. One teacher related, “I was the kind of teacher, I was very much temperamental and very restrictive, frightening students. Well, since I came to this workshop, … my temper dropped a little bit. … I have learned a lot … and I have made a new trend. So at this time now students can relate to me … and most of these students are now participating freely in my class (T1).” Teachers switched from perceiving student behavior as a threat to their authority, to perceiving their own behavior and lack of self-control as a threat to student learning. In the words of one teacher, “In the classroom, I’m not teaching student[s] to be afraid of me any longer, I’m helping them to learn (T7).” Teachers gave considerable thought to disciplinary processes, engaged student agency and remained flexible, a departure from teacher generated rules: “But nowadays, we … allowing students to have a role on issues. … But, at the same time too, we instructors who have the ability to think and rethink and be innovative in order- when you craft a law or ground rules in the class and you understand that the vast majority are not abiding by such a rule then as an instructor, you rethink and see how best you can craft another rule that you think can govern the class … So there are so measures as the teacher needs to put in place, in order for the student to govern itself (T1).” Teachers recognized that giving students more autonomy to buy into the materials or rewarding good behavior would decrease the need for the previously used teacher behaviors such as becoming angry, leaving the class or expelling students. “Because at first I never knew that, beating on child or … let’s say whenever the child having to do a positive thing, you give them … let’s say employ a reward, I never knew that, but from this training, I get to know that (T8).” They empowered students to contribute to class through questioning and discussions (see above). Teachers endeavored to make the material less frightening and more manageable. The shift from a focus on control to a focus on success for all required flexibility, openness and emotional awareness.

Some teachers expressed difficulty in figuring out how to motivate and engage students after using the previously favored harsh discipline: “If somebody disturbs a class and you tell that person ‘Stop disturbing!’ and that particular student knows fully well that he or she is not [going to be] whipped … or … beaten …, he will always disturb. … We tried to design some strategies in order to curtail that. So we are working on it and gradually we are getting there (T1).” Designing new methods for classroom control took effort and concentration. Teachers were willing to analyze their situations and adapt fluidly. Going from harsh punishment to positive behavioral systems generally results in less immediate results and requires more up-front investment on the part of the teacher. This is even more difficult if a negative relationship had already been established: “because I’m not the beating type now, they find it so easy to rile (T5).” The process of implementing non-violent discipline with students previously abused or witness to abuse in school was challenging for the teachers. Encouragingly, the interviewed teachers indicated that they would persist in modeling appropriate ways to manage emotions.

## Discussion

Participants in both Tier I and Tier II trainings gained knowledge of brain plasticity and neuroscience, an objective outcome. As measured by the survey scales, the training may have significantly decreased Tier I, but not Tier II participants’ subjective, negative attitudes and stigma toward people with MH problems. Some opinions from Tier II teachers tended to move in the correct direction after the training, only to rebound to more negative levels by the Refresher meetings. The interviewed Tier II teachers, however, described many positive changes to their practices that may have resulted from their new understanding of synaptic plasticity, adolescent brain development and the biological basis of mental illness. Notably, teachers reported reigning in their own negative emotional reactions in response to disruptive behaviors and working hard to build supportive relationships with students with social, emotional or behavioral problems. These personal accounts suggest the tiered training structure may have raised awareness of MH issues and influenced participants to re-evaluate classroom practices.

Attitude changes were registered among the Tier I cadre, but less so among the Tier II cadre. The difference in content or duration of the two trainings may have accounted for this. At the time of the Refreshers, Tier II participants’ reported more negative attitudes on the General Perceptions Scale. Many programs emphasizing education or MH literacy have resulted in more negative or absence of large, meaningful attitude changes or stigma reduction, despite successfully shifting acceptance of MI as biologically based diseases (Pescosolido et al., 2010; Corrigan et al., 2012; Stuart, 2016). More successful general audience stigma-reducing interventions utilize a social contact model where previously ill individuals recount their stories and recovery (Corrigan et al., 2012; Stuart, 2016). In comparison with MH literacy programs for teachers that emphasize behavioral signs, symptoms and appropriate responses (Anderson et al., 2019; Yamaguchi et al., 2020), neuroscience training alone may be insufficient to alter stigma. However, prior programs did not necessarily produce behavioral change toward persons with MH issues (Stuart, 2016; Anderson et al., 2019; Yamaguchi et al., 2020). Only one program in Tanzania reported an increase in referrals for professional help (Kutcher et al., 2016). In contrast, in response to the neuroscience emphasis of the current program, teachers reported changing disciplinary behaviors and support for students’ social and emotional needs. Thus, addition of the foundational knowledge provided by neuroscience to the behavioral focus of MH literacy programs may produce deeper learning and stronger behavioral change among teachers.

Key elements of programs aimed at reducing stigma toward MI among health care professionals include using multiple forms of message delivery, an emphasis on the ability to recover, teaching skills and behaviors for how to engage with people with MI, personal testimony and contact, myth-busting, and an enthusiastic facilitator (Knaak et al., 2014). These approaches have been implemented in other Liberian programs for health care workers, police and people living with MI (Kohrt et al., 2015, 2018; Gwaikolo et al., 2017; Liberia Center for Outcomes Research in Mental Health, 2020). The neuroscience emphasis of the current program focused upon dispelling erroneous beliefs about MI using multiple participatory forms of delivering the content by enthusiastic facilitators and practice teaching. The neuroscience emphasis sought to dispel the regionally strong, difficult to displace beliefs in witchcraft or curses as causes, and build on the strengths of students with social, emotional and behavioral problems to succeed in school (Ideanacho et al., 2014; Gwaikolo et al., 2017). The message regarding the biological basis of epilepsy appears to have been well received, internalized, and possibly translated into helping behaviors. While interviewees appeared to adopt the anti-stigma message, the overall social distancing scale results might have been improved if testimony from a recovered, affected individual had been included (Knaak et al., 2014; Thornicroft et al., 2016). Absence of sustained changes after anti-stigma program intervention, as observed here, is common, sometimes for lack of long term evaluation, but also because permanently changing attitudes and opinions remains a tenacious problem (Altindag et al., 2006; Uys et al., 2009; Thornicroft et al., 2016). Fully successful anti-stigma training programs are rare and should be culturally appropriate (Heim et al., 2019). The social burden from stigma may exceed that of the primary disease morbidity and mortality (Thornicroft et al., 2016), yet few programs effectively reduce multiple measures of stigma (Rao et al., 2019).

Disruptive student behaviors and a lack of positive teacher–student relationships contribute to teacher burnout (Skaalvik and Skaalvik, 2017). While the neuroscience training qualitatively may have impacted reported disciplinary practices and teacher–student relationships, it did not appear to alter teacher burnout. Given the overextended expectations for Liberian teachers (Adebayo, 2019), ameliorating this measure may have been overly hopeful. Surprisingly, burnout measures increased between the end of the Tier II trainings (fall 2018) and the Refresher time points (spring 2019). During this period, the Liberian economy shrank, inflation soared and the currency was devalued (African Development Bank, 2020). Teachers’ pay was delayed or not received, a common occurrence (Adebayo, 2019; Johnson, 2019; Romero and Sandefur, 2019), resulting in strikes and student protests (Dunbar, 2019). Thus teachers had ample reasons outside the trainings to feel their work in schools had not been valued. Future studies should track such external systemic factors for their influence upon outcomes.

As teachers reflected that the training program appeared to influence their approach to discipline, this training program may have produced a positive impact on teachers’ disciplinary behavior. Future studies would benefit from including objective measures to examine the impact of the program on this outcome. This is of particular importance given the negative impact that classroom discipline can have on students and their ability to learn – a point that teachers’ comments suggested they now appreciate. Corporal punishment of children is a widespread normative practice in Liberia, as in many countries worldwide, among parents, teachers and caregivers (Antonowicz, 2010; Gershoff, 2017; EACPC, 2018). In accordance with ratification of the UN Convention on the Rights of Children in 1993, the Liberian Teacher Code of Conduct specifically prohibits both physical and verbal abuse (Ministry of Education, 2014). The 2010 National Education Sector Plan contained wording establishing that for all ages of children and youth, a school environment should be “clean, sanitary, violence-free and sufficiently conducive for all students, especially girls, to feel safe and at ease.” (Ministry of Education, 2010; p. xii–xiv). Experiencing corporal punishment has been separately linked to poorer math test scores 4 years later and greater mental and behavioral problems (Jones and Pells, 2016; Gershoff, 2017). While not directly designed to address issues of interpersonal or societal violence, the program clearly resulted in behavioral changes that dialed down the level of disciplinary violence in interviewees’ classrooms. Training in Uganda using the Good Schools Tool kit, developed to address violence toward children emanating from school staff, significantly reduced student reported corporal punishment after 18 months of implementation (Devries et al., 2015). Our intervention only used one exercise from this program to lead teachers in identifying the causes of student misbehavior (see section 3.5^1^) (Devries et al., 2015). Tier II teachers did, however, engage in animated discussions surrounding how to apply knowledge gained in the training to their classrooms. Simply understanding that adolescent brains and associated decision-making capability are not fully developed and therefore not equivalent to an adult’s capacity, provided teachers with a strong reason to use patience in student interactions and in assessing their responses. Understanding that thinking takes time convinced teachers to pause and wait for student answers rather than rapidly punishing them for not responding.

Physical punishment, at odds with “stated” national educational policy, is common in Liberian schools (Antonowicz, 2010; Gershoff, 2017; EACPC, 2018). Interviewed teachers discussed and largely admitted to metering out harsh physical punishment as disciplinary practice. However, none of the interviews referred to gender-based violence toward students. During the Refresher discussion, several male teachers said what they learned during the training about the traumatic impact of engaging in sexual activities with students led them to cease doing so. School-related gender-based violence has been documented in Liberian schools in the aftermath of the civil war and continues into the present (Parkes, 2016). In one survey of secondary students from four Liberian counties, 43% of students reported being sexual coerced and 91% reported experiencing gender-based violence (Postmus et al., 2015). Males and females were equally at risk. Notably 27% of males and 40% of females reported having been asked for or engaged in sex as part of a transaction for better grades, school uniforms or fees, food, or money (Postmus et al., 2015). Transactional sex leads to increased social status among peers and is sometimes supported by parents as it provided a means to an education (Atwood et al., 2011; Postmus et al., 2015). Such practices persist despite the Ministry of Education 2014 Teacher Code of Conduct (Ministry of Education, 2014; Steiner et al., 2018). Considering this cultural context, statements made in Refreshers demonstrated courage, group trust, and a real commitment to change on the part of the participants sharing this information. While the neuroscience content focused upon the connections between neurophysiological stress responses and learning, the Tier II workshop content adaptations which included discussions of practices in participants’ classrooms may ultimately contribute to progress on this intractable problem. These discussions demonstrate the clear benefit of appropriate cultural responsiveness and adaptation of the training to local conditions (Southall et al., 2017; Steiner et al., 2018; McCallops et al., 2019).

Trauma-informed policies and recommendations appropriately address the needs of individuals with a history of trauma and students in particular (Cole et al., 2009; Fallot and Harris, 2009; NCTSN Core Curriculum on Childhood Trauma Task Force, 2012; Trauma and Justice Strategic Initiative, 2014). For trauma-informed teaching, schools should *realiz*e the impact associated with prior trauma, *recognize* signs of trauma among students, *respond* in a sensitive, integrated manner, and actively prevent *re-traumatization* (Trauma and Justice Strategic Initiative, 2014). The current program may have built a *realization* of how various traumas can impact students both acutely and chronically. Teachers attested that they could *recognize* signs of mental stress among students and *respond* to those needs. Lastly, teachers actively changed their own disciplinary practices to *prevent* further exclusion and traumatization of the students. Additional guidelines proposed teachers build secure relationships with students, enhancing self-regulation, and increasing competencies (NCTSN Core Curriculum on Childhood Trauma Task Force, 2012). Tier II teachers talked extensively about building relationships with students who they formerly would have ignored. Moreover, teachers reported changing their practices to include activities that provided students with the agency to become involved in their own learning and to grow their autonomy (Brick et al., 2021). Values associated with trauma-informed care include safety, trustworthiness, choice, collaboration and empowerment (Fallot and Harris, 2009; Trauma and Justice Strategic Initiative, 2014). The student-centered pedagogies enacted by the interviewed teachers may have empowered students by giving them opportunities to collaborate with peers in group settings and exercise choice in active learning exercises. In working toward stronger relationships with their students, teachers reported building student trust in their ability to relate to the teacher, the school and to their own learning. By suspending harsh disciplinary practices, teachers reported shifting the power dynamic and created safer learning environments. Thus, the combination of neuroscience and MH topics covered many trauma-informed teaching concepts and may have produced improvements in teacher behavior consistent with these ideas.

### Limitations

Two technical issues limit the interpretation of this pilot study. First, the qualitative and follow-up data collection occurred among the Tier II and not the Tier I teachers, preventing comparisons at the extended time point between the cadres. Second, the Tier II teachers responded to the surveys at three times, prior to and at the end of the workshop and again at the Refresher training, whereas Tier I teachers only responded to the survey at the single end-of workshop time point. In doing so, Tier I teachers’ new knowledge may have allowed them to assess their positions on the various scales currently and more accurately state what their positions were prior to the training. This retrospective view acknowledges what has been learned and does not overvalue naïve initial conceptualizations (Levinson et al., 1990; Bhanji et al., 2012). If recalibration to a new normal becomes established as a result of the training, follow-up responses might fail to register change. Employing a retrospective survey might ensure that a respondent did not recalibrate their responses between initial training and follow-up.

Several other limitations impact interpretation of the current findings and should be addressed in subsequent endeavors. The non-randomized, uncontrolled nature of this pilot study means that the current model and results cannot be generalized without more rigorous testing using a randomized experimental design with control groups. The ability of the neuroscience content to motivate behavioral teacher changes should be compared to MH training without a neuroscience component. Individual teachers were targeted for the training, placing a burden on them to implement and translate the new ideas to their own schools, where local culture works strongly against change. Recruitment to the current program by heads of schools followed local customs but did not produce a random sample of teachers, which may have introduced a selection bias. Different findings may emerge among training participants who are self-selected. For larger tests of this model, randomization by schools and inclusion of controls should be considered. In that way, future programs could also target administrators and teacher cadres from the same schools to provide communities of local support for continued application of workshop ideas (Devries et al., 2015). As secondary science teachers, workshop participants were among the most educated group of teachers in Liberia. Spaced trainings with more behavioral support might be needed to similarly impact the majority of Liberian teachers. Follow-up data collection focused only on teacher attitudes and self-reported practices. We were unable to document if teachers indeed conveyed neuroscience ideas to learners. Future teacher trainings should identify and examine effects upon pedagogy, classroom environment, and student opinions, performance and resilience, measures that go beyond self-report. In addition, measures should be adopted to probe in a more structured manner the themes that emerged from the qualitative analysis; corporal punishment, disciplinary actions and transactional sex.

### Recommendations and Conclusion

The positive behavioral changes reported from this pilot program merit more rigorous testing and reproduction on a larger scale. Efforts to train more teachers may require dedicated teacher coaches, government action and administrative structure (Gove et al., 2017). Future programs should consider strengthening the combined neuroscience and MH literacy program through inclusion of people with lived MI experiences as co-trainers (Corrigan et al., 2012). More practice using positive behaviors for handling discipline problems would be helpful. Confidentiality, tactfulness, and abuse are among additional topics that could be included. The program did not emphasize, but perhaps should include discussion surrounding placing teachers’ responsibilities and responses within the boundaries of confidentiality. Teachers requested inclusion of more content regarding the effects of neuroactive drugs, more dissections, more practicums and more collaborative discussions. Addressing an audience of administrators and groups of teachers from the same schools would create opportunities for change throughout the educational system. Continuing the training, spaced over multiple years, would strengthen teachers’ own learning and work to build strong communities of practice. The Training-of-Trainers model was feasible and potentially more cost effective than the privatization of entire schools for implementing pedagogical reforms (Romero and Sandefur, 2019).

This pilot study provides initial evidence that combining basic neuroscience and MH training may bring about behavioral changes among secondary science teachers in a LMIC. The key neuroscience concepts covered included brain plasticity, adolescent brain development, how stress and emotional states modulate learning, and the etiology of epilepsy and MH problems in Liberia. The key MH information focused on how to recognize and refer students with social, emotional, behavioral and mental health issues. The qualitative evidence pointed to apparent changes among these particular teachers in understanding of students MH and efforts to build positive relationships with students. Helping behaviors and overall social and emotional support appeared to increase, both for MH issues and normal learning. Reported use of abusive behaviors by participant teachers decreased. These qualitative behavioral changes may be the more important and lasting outcomes. Whether or not such changes could be generalized to a larger group of teachers cannot be determined without further program modification and replication.

## Data Availability Statement

The raw data supporting the conclusions of this article will be made available by the authors, without undue reservation.

## Ethics Statement

The studies involving human participants were reviewed and approved by Institutional Review Boards of Emory University and the University of Liberia. The patients/participants provided their written informed consent to participate in this study.

## Author Contributions

KB managed the program, developed curriculum, delivered the Tier II trainings, curated, analyzed, and interpreted data, and edited the manuscript. JLC conceived, designed, and led the program and evaluation, developed curriculum, analyzed and interpreted data, and edited the manuscript. LM and SF managed the program, developed curriculum, and delivered the Tier II trainings. JM curated and analyzed quantitative data. JMD designed and delivered the curriculum and Tier I trainings, provided the neuroscience content, analyzed and interpreted data, wrote and edited the manuscript. All authors contributed to the article and approved the submitted version.

## Conflict of Interest

The authors declare that the research was conducted in the absence of any commercial or financial relationships that could be construed as a potential conflict of interest.

## Footnotes

1 raisingvoices.org
