# Supplementary material for: Training-of-Trainers Neuroscience and Mental Health Teacher Education in Liberia Improves Self-Reported Support for Students
Source: Front Hum Neurosci. 2021 Jun 18;15:653069. doi: 10.3389/fnhum.2021.653069 (PMC8249721; doi:10.3389/fnhum.2021.653069)
Supplement: Supplementary file 1 [file Data_Sheet_1.PDF]

Supplementary Materials

The Carter Center Mental Health Program in Liberia
Neuroscience Training for Liberian Educators Project
Post-Training Interview Guide

Demographics

County: _____________ Age: _______________

Sex: _______________ Role: ____________________________

Questions

1) How do you describe your role as a teacher?

2) How do you determine individual students' needs?

3) Every class has really fast learners, normal learners, and learners who lag behind. How do you teach to each set of learners?

4) What behaviors would lead you to think a student might be having mental health problems?

5) Once you recognize behaviors suggesting mental health problems, what do you do for that student?

6) What is your opinion of the BrainU training?
    a. Has it helped you in your job? If so, how has it helped you?
    b. Has it made aspects of your job more difficult? How has it made it more difficult?

7) What are some skills you learned from the training?

8) What (if any) changes have you implemented in your teaching since the training?

9) What challenges have you faced in trying to implement what you learned in the training?

10) What successes have you had in supporting students with social, emotional, behavioral, or mental health problems?

Supplementary Table 1. Descriptive statistics for survey data depicted in Figs. 2, 3 and 4.

Scale	Tier	When	Mean	SD	N	Range
**Social Distance**	I	Retrospective Pre	24.4	6.3	23	11-35
		Post	19.7	6.9	24	9-33
	II	Pre	23.7	7.1	44	10-36
		Post	20.2	9.0	40	9-36
		Refresher	23.9	5.7	46	16-36
**General Perceptions**	I	Retrospective Pre	14.9	0.9	22	8-24
		Post	10.9	0.6	23	7-15
	II	Pre	14.2	3.1	44	9-22
		Post	12.3	3.9	38	7-22
		Refresher	15.6	2.5	56	11-22
**Maslach Burn Out**	I	Retrospective Pre	46.6	22.9	23	13-84
Full scale		Post	34.8	19.1	24	1-69
	II	Pre	41.5	15.0	43	17-74
		Post	42.8	15.2	39	22-74
		Refresher	51.0	13.0	56	29-78
2 question scale	I	Retrospective Pre	4.5	3.6	23	0-12
		Post	3.5	2.9	24	0-10
	II	Pre	6.2	2.6	43	0-12
		Post	6.7	3.6	39	0-12
		Refresher	7.1	3.6	56	0-12
Emotional Exhaustion subscale	I	Retrospective Pre	22.8	14.3	23	1-53
		Post	17.2	2.3	24	1-39
	II	Pre	22.9	10.3	43	0-41
		Post	22.6	8.3	39	0-42
		Refresher	27.0	8.3	56	11-43
Personal Accomplishments	I	Retrospective Pre	13.0	9.8	23	1-34
		Post	11.0	8.4	24	0-35
	II	Pre	8.0	5.1	43	0-21
		Post	8.5	7.9	39	0-26
		Refresher	9.4	7.6	56	0-25
Depersonalization	I	Retrospective Pre	10.8	8.4	23	0-30
		Post	6.6	5.1	24	0-18
	II	Pre	10.7	6.6	43	0-28
		Post	11.7	5.9	39	1-27
		Refresher	14.7	5.3	56	5-27
